# ALK-rearranged and EGFR wild-type lung adenocarcinoma transformed to small cell lung cancer: a case report

**DOI:** 10.3389/fonc.2024.1395654

**Published:** 2024-04-23

**Authors:** Rui Chen, Yan Jian, Yuzhen Liu, Junping Xie

**Affiliations:** ^1^ Department of Respiratory and Critical Care Medicine, The Second Affiliated Hospital, Jiangxi Medical College, Nanchang University, Nanchang, Jiangxi, China; ^2^ Jiangxi Provincial Cancer Hospital, The Second Affiliated Hospital of Nanchang Medical College, Nanchang, Jiangxi, China; ^3^ Graduate School, Jiangxi Medical College, Nanchang University, Nanchang, Jiangxi, China

**Keywords:** non-small cell lung cancer, small cell lung cancer, transformation, ALK-TKIs, liver metastasis

## Abstract

**Background:**

Cases of ALK-rearranged EGFR wild-type lung adenocarcinoma (LUAD) transforming into small cell lung cancer (SCLC) are rarely reported, and diagnosis is often delayed. The emergence of this transformation phenomenon is often regarded as a consequence of acquired resistance mechanisms.

**Case presentation:**

A 47-year-old male diagnosed with poorly differentiated adenocarcinoma of the right middle lung (pT2N2M0, stage IIIA) achieved a 46-month progression-free survival (PFS) following surgery and adjuvant chemotherapy. During routine follow-up, tumor recurrence and metastasis was detected. Genetic testing revealed ALK rearrangement and wild-type EGFR, prompting treatment with ALK-TKIs. In May 2023, abdominal CT scans showed significant progression of liver metastases and abnormal elevation of the tumor marker NSE. Immunohistochemical results from percutaneous liver biopsy indicated metastatic SCLC.

**Results:**

After resistance to ALK-TKIs and transformation to SCLC, the patient received chemotherapy combined with immunotherapy for SCLC, but the patient’s disease progressed rapidly. Currently, the patient is being treated with albumin-bound paclitaxel in combination with oral erlotinib and remains stable.

**Conclusion:**

Histological transformation emerges as a compelling mechanism of resistance to ALK-TKIs, necessitating the utmost urgency for repeat biopsies in patients displaying disease progression after resistance. These biopsies are pivotal in enabling the tailor-made adaptation of treatment regimens to effectively counteract the assorted mechanisms of acquired resistance, thus optimizing patient outcomes in the battle against ALK-driven malignancies.

## Introduction

Chromosomal rearrangements within the Anaplastic lymphoma kinase (ALK) gene play a pivotal role in determining the heightened sensitivity of a subset of non-small cell lung cancers (NSCLC) to small molecule ALK tyrosine kinase inhibitors (ALK-TKIs) (1). As per guideline recommendations, targeted therapy has now become the primary standard of care for patients with locally advanced and metastatic ALK-positive NSCLC (2). The advent of ALK-TKIs, including first-generation crizotinib, second-generation alectinib, brigatinib, and ensartinib, as well as third-generation Lorlatinib, has significantly revolutionized treatment choices and prognosis for individuals with ALK-positive NSCLC. Nevertheless, it is essential to acknowledge the inevitability of drug resistance alongside challenges like distant metastasis, the potential for severe adverse effects, and a diminished quality of life encountered during therapy (3). Common drug resistance mechanisms include secondary mutations in ALK, amplification of ALK fusion gene copies, and activation of bypass and downstream pathways (4). While ALK-TKIs are still being updated and iterated to address issues such as drug resistance, the silent phenomenon of SCLC transformation does not appear to have received much attention.

Indeed, this transformation from NSCLC to SCLC occurs mostly in patients resistant to EGFR-TKIs (5–7). This transformation has been described as a mechanism of acquired resistance occurring in approximately 5% of patients who develop resistance to EGFR-TKIs (8). Despite case reports, transformation from NSCLC to SCLC due to resistance to ALK-TKIs remains a rare phenomenon.

We present a case of a patient with NSCLC who developed recurrence and metastasis after surgery, progressed and transformed to SCLC after being resistant to treatment with ALK-TKIs. Subsequent application of therapy targeting SCLC led to transient control of the disease. The current treatment regimen is albumin-bound paclitaxel in combination with anlotinib, and the patient’s condition is still stable.

## Case report

**Figure 1:**
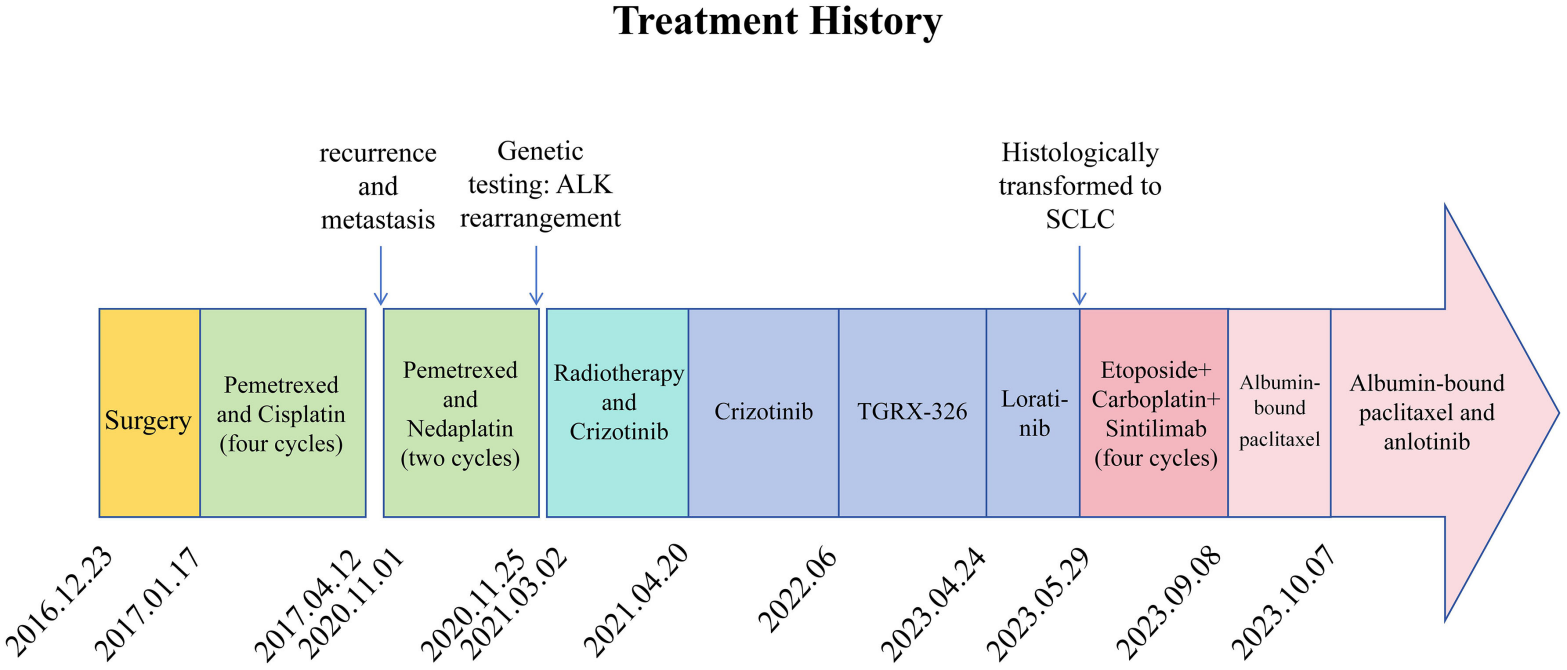
Patient’s treatment history and medication details.

A 47-year-old male patient with a smoking history was admitted for evaluation of mild discomfort in the right chest and back on 30th November 2016. Physical examination revealed that the patient had diminished breath sounds and audible rales in the right middle lung. Chest CT revealed a nodular shadow near the hilum of the right middle lobe, suggesting a high possibility of lung cancer with enlarged mediastinal lymph nodes. The patient had mild hypertension, no other diseases and no family history of tumors. After excluding contraindications for surgery, the patient underwent video-assisted thoracoscopic surgery for right middle lobe resection and mediastinal lymph node dissection on 23rd December 2016. Postoperative pathology revealed poorly differentiated adenocarcinoma of the right middle lung (pT2N2M0, stage IIIA), with positive immunohistochemical markers for CK, CK7, TTF-1, CgA, and Ki67 (40% positive staining) (**Supplementary Figure 1**). Starting from 17th January 2017, the patient received four cycles of post-operative adjuvant chemotherapy using the PP regimen (Pemetrexed 500mg/m^2^ and cisplatin 75mg/m^2^ once every three weeks) (specific treatment process illustrated in **Figure 1**), followed by regular outpatient follow-ups. The patient achieved a PFS of 46 months postoperatively.

**Figure 2:**
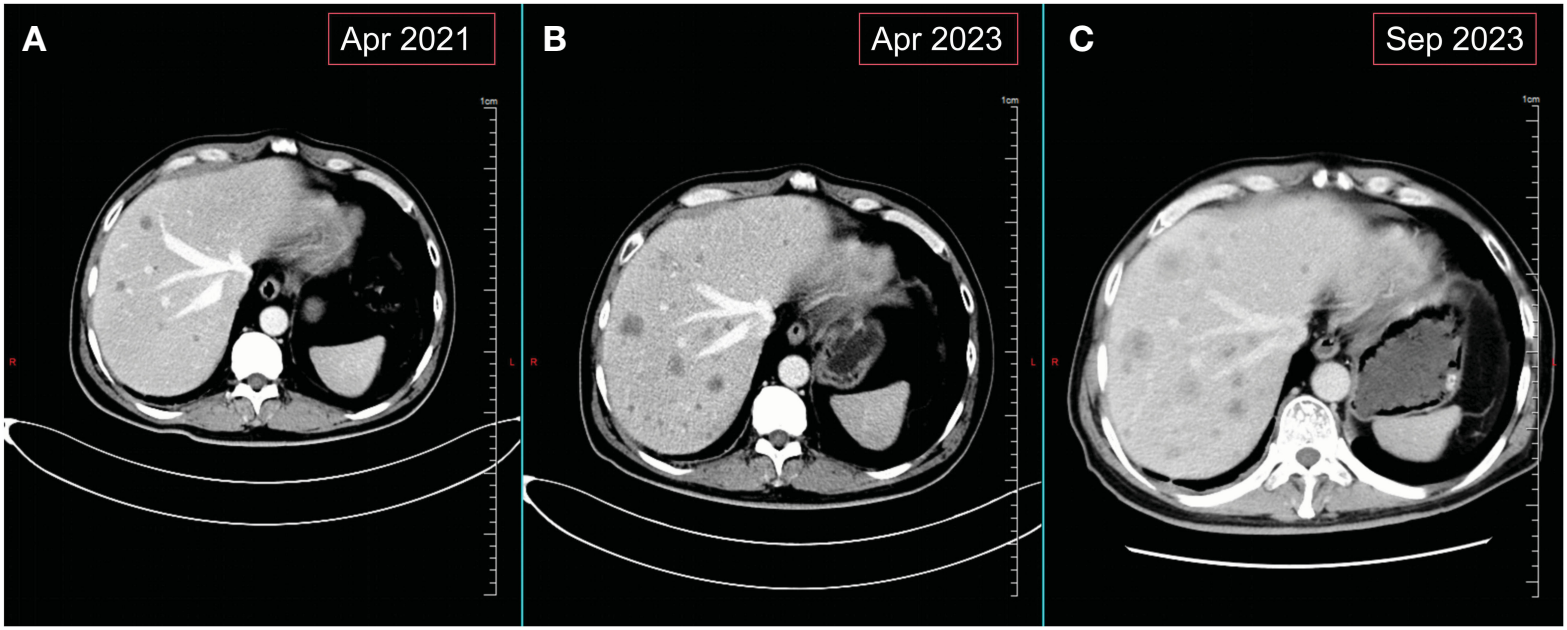
Changes in liver metastases on abdominal CT. **(A)** Images of liver metastases before monotherapy with crizotinib. **(B)** Images of the liver at the time of disease progression following treatment with TGRX-326. **(C)** Hepatic imaging of disease progression following use of 4 courses of chemotherapy combined with immunotherapy for SCLC.

In early September 2020, the patient experienced occasional hemoptysis. On October 19, 2020, a follow-up chest CT revealed slightly increased multiple nodules in the mediastinum compared to previous findings (**Supplementary Figures 2A, B**). An upper abdominal MRI indicated hepatic metastases and enhanced lesions in the lumbar vertebrae, suggesting bone metastasis (**Supplementary Figure 2C**). These findings suggested tumor recurrence and metastasis in the patient, prompting us to conduct genetic testing on the patient (Jinyu Medical Laboratory Center, Guangzhou, China). Due to the time required for waiting for genetic testing results, considering the progression of the patient’s condition and the patient’s desire for treatment, systemic chemotherapy was administered as the first step. Starting from November 1, 2020, the patient received two cycles of chemotherapy, including pemetrexed 500mg/m^2^ and Nedaplatin 80-100mg/m^2^ once every three weeks. After chemotherapy, patients exhibited poor tolerance, characterized by Grade II gastrointestinal adverse reactions and significant hematological adverse events. After aggressive symptomatic treatment, the patient’s symptoms were partially relieved. However, this led to a slight delay in subsequent treatment. Concurrently, genetic testing revealed the patient’s epidermal growth factor receptor (EGFR) mutation to be negative, while harboring an ALK rearrangement. At the beginning of 2021, the patient presented with lower back pain. Physical examination revealed tenderness in the lumbar region, without evidence of spinal deformities or signs of neural involvement. Considering the patient’s symptoms and lumbar spine MRI findings (**Supplementary Figure 2D**), we considered that the lumbar pain is induced by bone metastasis. From March to April of the same year, palliative radiotherapy was administered to the L4 metastatic lesion in the lumbar spine at a dose of 39Gy in 13 fractions, whole-brain radiotherapy at a dose of 30Gy in 15 fractions, and a local boost of 12Gy in 6 fractions. The patient’s symptoms improved (additional brain metastasis images from the patient can be seen in **Supplementary Figure 3**). Since genetic testing revealed the presence of an ALK rearrangement, the patient received oral crizotinib at a dose of 250 mg twice daily after radiotherapy and achieved a PFS of 15 months. In June 2022, a follow-up upper abdominal MRI showed progression of hepatic lesions compared to previous findings. Patients opted into the clinical trial after giving full informed consent. The patient’s treatment was switched to TGRX-326 (a third-generation ALK/ROS1 tyrosine kinase inhibitor) at a dose of 60 mg once daily to continued targeted therapy, with disease stability (SD) as the assessed treatment response, until April 24, 2023. The patient achieved a PFS of 11 months. Subsequently, progressive disease (PD) was observed (**Figures 2A, B**), leading to the patient’s withdrawal from the clinical trial. Treatment was then switched to oral administration of the third-generation targeted therapy drug loratinib, at a dosage of 100mg once daily.

**Figure 3:**
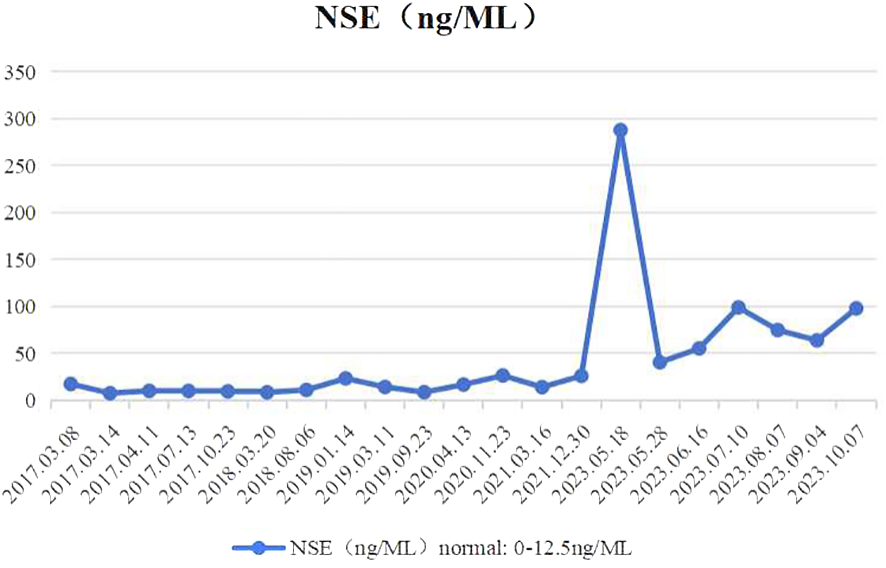
The profile of NSE in serum.

**Figure 4:**
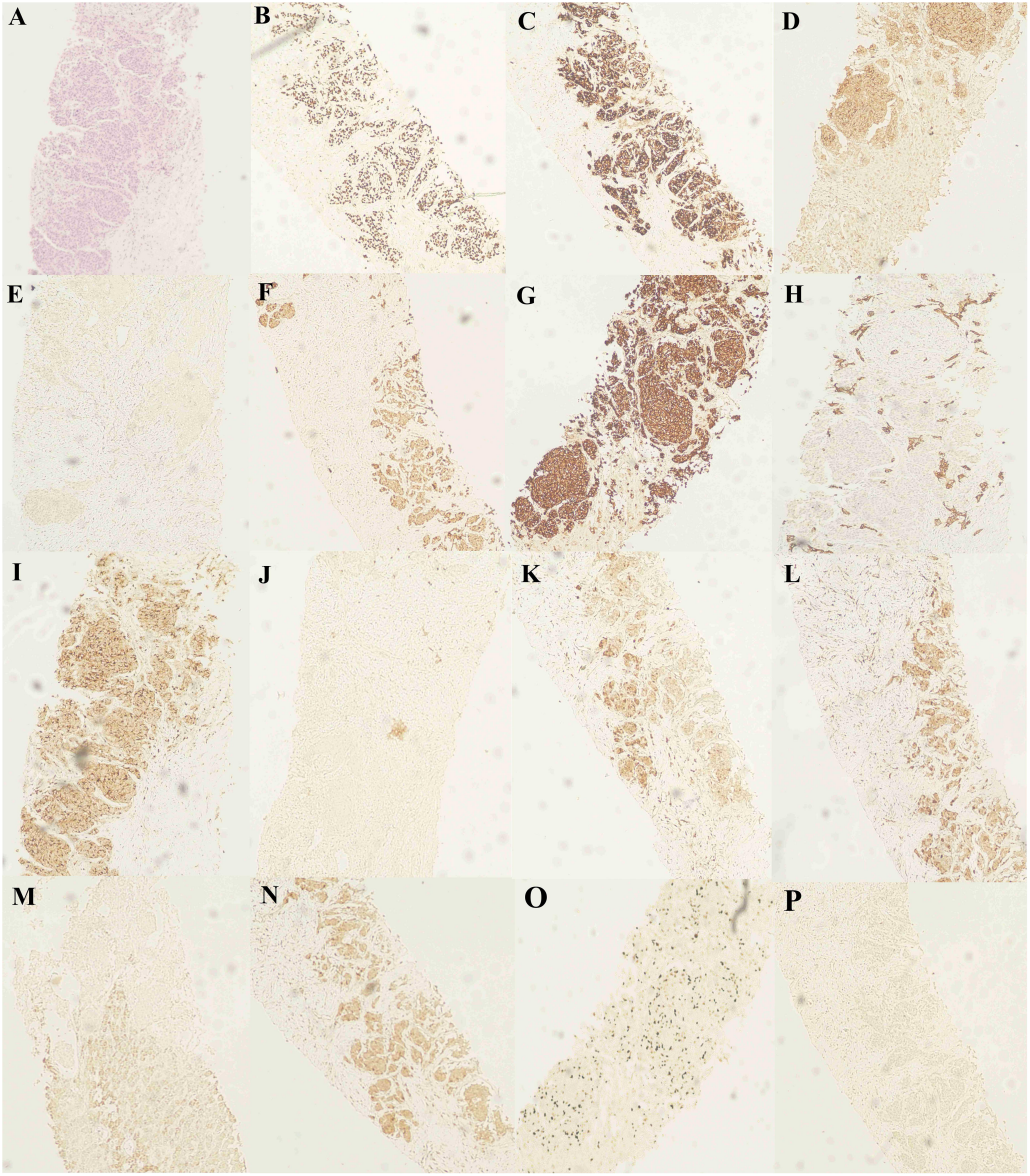
Hematoxylin and eosin (H&E) staining and immunohistochemical staining (IHC) staining of the liver biopsy specimen. **(A)** H&E staining of the liver biopsy specimen. **(B-D)** IHC staining confirmed positive for TTF-1, CgA and CD56. IHC staining confirms **(E)** AFP-negative and **(F-H)** CK, CK7 and CK19-positive. IHC staining confirmed **(I)** CD10 positivity, **(J)** Napsin A negativity, **(K)** GPC-3 positivity, and **(L)** GS positivity. IHC staining confirmed **(M)** HepPar-1 negativity, **(N)** HSP70 positivity, **(O)** Ki-67 positivity, and **(P)** P40 negativity.

Patient revisited on 2023-05-22 and was found to have a high level of neural-specific enolase (NSE), a tumor marker, reaching 287.6 ng/mL (**Figure 3**). Percutaneous liver biopsy was performed, and pathological examination revealed metastatic small cell carcinoma, most likely from the lungs. Immunohistochemistry results showed: CD56 (+), CgA (+), broad-spectrum CK (+), P40 (-), TTF-1 (+), Ki-67 (+, 40%), CK7 (+), Napsin A(-), CK19 (+), CD10 (+), AFP (-), GPC-3 (+), HSP70 (+), GS (+), HepPar-1 (-), Syn (+) (**Figure 4**). Therefore, we transitioned the treatment protocol to target SCLC. Starting from 2023-05-29, as palliative first-line treatment following transformation of the pathological type, the patient received treatment with 100mg/m^2^ of etoposide on days 1-3, carboplatin injection (AUC of 5 mg/ml/min) on day 1, and 200mg of sintilimab on day 1, once every three weeks, for a total of 4 cycles. After treatment, NSE was significantly reduced compared to before and the patient achieved a PFS of 3 months. On 2023-09-06, follow-up chest and abdominal CT scans indicated increased lymph node metastasis in the mediastinum and multiple liver lesions compared to previous scans (**Figure 2C**). In light of the deterioration in the patient’s condition, we opted to switch to paclitaxel-based medication as palliative second-line treatment for SCLC. Starting on 8 September 2023, patients received a single course of systemic chemotherapy with intravenous albumin-bound paclitaxel. Nevertheless, the patient’s tumor marker NSE continued to rise. Considering the patient’s satisfactory physical condition (with a PS score of 2) and tolerance, a combined approach with anlotinib targeted therapy was initiated. On October 7, 2023, the patient was readmitted for treatment and received intravenous administration of albumin-bound paclitaxel at a dose of 260mg/m^2^ every 3 weeks, along with concurrent oral administration of anlotinib at a daily dosage of 8 milligrams, administered for two weeks followed by a one-week break. After treatment, the patient’s mental state and appetite were satisfactory and his condition was stable (see **Supplementary Table 1** for detailed patient information).

## Discussion

Lung cancer is the leading cause of cancer-related deaths globally, with NSCLC accounting for approximately 80-85% of lung cancer cases (9). Up to 8% of NSCLC patients have an ALK rearrangement, most commonly a fusion between ALK and the echinoderm microtubule-associated protein-like 4 (EML4), resulting in an EML4-ALK fusion that drives continuous cell proliferation and ultimately tumor formation (10). Crizotinib is currently the standard first-line therapy for ALK-positive NSCLC patients. However, due to various resistance mechanisms, most patients experience relapse within one year of treatment. The Phase III CROWN Study demonstrated that compared to the first-generation crizotinib, the third-generation ALK-TKI lorlatinib can improve progression-free survival (PFS) and reduce central nervous system progression in patients with advanced ALK-positive NSCLC (11). Nonetheless, drug resistance remains inevitable, with the most common mechanism being drug-resistant mutations in ALK. Case report have demonstrated that the NSCLC patient with ALK fusion mutations developed ALK fusion V1180L mutation and transformed into SCLC after acquiring resistance to alectinib (12). And, there was also a LUAD patient who developed ALK G1202R mutation and SCLC transformation after treatment resistance to second-generation ALK-TKIs (13). Although these are rare case reports, they carry significant implications. The poor response of patients to ALK-TKIs not only suggests the potential emergence of drug-resistant mutations but also necessitates consideration of the possibility of SCLC transformation.

In fact, cases of transformation from NSCLC to SCLC occurred mainly in patients with EGFR-mutated LUAD who were resistant to EGFR-TKIs (5–7, 14). To explore the molecular mutational mechanisms underlying this transformation, researchers have conducted next-generation sequencing (NGS) or whole-exome sequencing (WES) on samples from LUAD patients who transformed into SCLC after developing resistance to EGFR-TKIs. Multiple studies have consistently shown that this histological transformation is closely associated with inactivation mutations in the Retinoblastoma1 (Rb1) and TP53 genes, indicating that the inactivation of Rb1 and TP53 is an effective predictive factor for the transformation of LUAD into SCLC (7, 8, 15–18). Additionally, researchers reported a case of RET-rearranged LUAD transforming into SCLC, with acquired resistance to pralsetinib. Molecular analysis revealed the presence of the same RET fusion and TP53 mutation in the primary LUAD and recurrent SCLC (19). Previously, a patient with ALK-rearranged NSCLC experienced disease progression after treatment with ALK-TKIs, followed by SCLC transformation. Genomic profiling revealed the retention of ALK rearrangement, accompanied by inactivating Rb1 mutation (C706Y) and p53 exon deletion, which were not detected in the original tumor tissue at diagnosis (13). This also provides evidence supporting the significant roles of p53 and Rb1 loss in SCLC transformation. Additionally, other mutations possibly associated with transformation include PIK3CA mutation, WNK1 mutation, etc. (20, 21). Furthermore, research suggests that the presence of neuroendocrine differentiation in NSCLC may be one of the factors leading to SCLC transformation (22). The origin of this transformation from NSCLC to SCLC is a controversial topic, as it is not entirely clear whether the original lung cancer tissue harbored mixed components (23).

Transformed SCLC typically manifests rapid disease progression and poses therapeutic challenges. Currently, there is no standardized treatment strategy for patients who develop SCLC transformation after resistance to ALK-TKIs. Chemotherapy combined with immunotherapy is the most common treatment option. In the entirety of this patient’s therapeutic journey, the regimen devised was personalized, integrating the patient’s actual condition while adhering to treatment norms. For instances, following the pathological transformation to SCLC, commonly used treatment options carboplatin, etoposide, and atezolizumab or durvalumab (24, 25), were not selected; rather, carboplatin, etoposide, and sintilimab were chosen. The choice of immunotherapeutic agents primarily stemmed from the patient’s limited financial capacity, unable to afford imported PD-L1 inhibitors, thus opting for domestically produced sintilimab in combination with chemotherapy, yielding a 3-month PFS. Indeed, studies have demonstrated that sintilimab can serve as maintenance therapy post-chemotherapy for SCLC (26). The combination of sintilimab and anlotinib as second-line or beyond therapy for extensive disease (ED)-SCLC exhibits favorable antitumor activity with manageable toxicity (27). Additionally, prior reports have shown the efficacy of sintilimab in ED-SCLC refractory to multi-line treatments (28). Therefore, we opted for this regimen. This case underscores the necessity, in clinical practice, to tailor treatment approaches to individual patients by considering treatment guidelines alongside factors such as the patient’s actual physical condition and economic status.

According to the literature, it has been pointed out that a rapid elevation of serum NSE and poor response to targeted drugs usually indicate a transformation from LUAD to SCLC (29). This trend was also observed in the present case, where a follow-up liver imaging examination after approximately 26 months of taking ALK-TKIs indicated disease progression and an elevated NSE level of 287.6ng/ML (normal range: 0-12.5ng/ML). This also suggests that during treatment, clinicians should monitor serum tumor markers or perform genomic sequencing, especially in patients with disease progression, as this may aid in the early detection of SCLC transformation. Repeat biopsies may be performed if necessary, and treatment plans can be adjusted promptly based on molecular pathological examination results to achieve personalized and comprehensive management of patients. Additionally, in this case, despite developing drug resistance and multiple metastases during the course of treatment, the patient’s survival time since the initial diagnosis has over seven years. In a certain sense, regular follow-up visits, improved doctor-patient communication, and enhanced patient compliance hold great practical significance in improving patient prognosis.

## Conclusion

This study reports a case of LUAD patient with postoperative recurrence and metastasis who developed acquired resistance and underwent SCLC transformation following treatment with ALK-TKIs. This finding has influenced clinical practice, highlighting the importance of dynamic assessment of tumor markers and repeat biopsies when necessary. The results suggest the necessity of early development of personalized treatment plans for patients experiencing SCLC transformation after ALK-TKIs resistance, with regular follow-up appointments and timely adjustment of treatment strategies advised. This subset of patients appears to exhibit faster disease progression compared to typical SCLC patients.

## Data availability statement

The original contributions presented in the study are included in the article/**Supplementary Material**. Further inquiries can be directed to the corresponding author.

## Ethics statement

Written informed consent was obtained from the individual(s) for the publication of any potentially identifiable images or data included in this article.

## Author contributions

RC: Investigation, Conceptualization, Data curation, Formal analysis, Visualization, Writing – original draft. YJ: Supervision, Writing – review & editing. YL: Supervision, Writing – review & editing. JX: Supervision, Writing – review & editing, Investigation.

## Supplementary material

Supplementary Figure 1 H&E staining and IHC staining of surgical resection specimens. **(A)** H&E staining of surgically excised specimens. **(B-D)** IHC staining of the surgically resected specimen showed positivity for TTF-1, CK, and Ki67.

Supplementary Figure 2 CT images of the patient’s chest and MRI images of the abdomen. **(A, B)** Mediastinal window of chest CT demonstrating the patient’s mediastinal lymph nodes. **(C, D)** Abdominal MRI images showed tumor invasion of the patient’s lumbar spine at L4.

Supplementary Figure 3 MRI image of the patient’s cranium. **(A)**. Cranial MRI images of the patient before whole brain radiotherapy. **(B, C)** are cranial MRI images of the patient after whole brain radiotherapy.

## References

[1] Shaw A Bauer TM de Marinis F Felip E Goto Y Liu G . First-line lorlatinib or crizotinib in advanced ALK-positive lung cancer. New Engl J Med. (2020) 383:2018–29. doi: 10.1056/NEJMoa2027187 33207094

[2] Ettinger D Wood D Aisner D Akerley W Bauman J Bharat A . Non-small cell lung cancer, version 3.2022, NCCN clinical practice guidelines in oncology. J Natl Compr Cancer Net. JNCCN. (2022) 20:497–530. doi: 10.6004/jnccn.2022.0025 35545176

[3] Katayama R . Drug resistance in anaplastic lymphoma kinase-rearranged lung cancer. Cancer Sci. (2018) 109:572–80. doi: 10.1111/cas.13504 PMC5834792 29336091

[4] Schneider J Lin J Shaw A . ALK-positive lung cancer: a moving target. Nat Cancer. (2023) 4:330–43. doi: 10.1038/s43018-023-00515-0 PMC10754274 36797503

[5] Liu Y . Small cell lung cancer transformation from EGFR-mutated lung adenocarcinoma: A case report and literatures review. Cancer Biol Ther. (2018) 19:445–9. doi: 10.1080/15384047.2018.1435222 PMC5927699 29461911

[6] Ren X Cai X Li J Zhang X Yu J Song X . Histological transformation of lung adenocarcinoma to small cell lung cancer with mutant C797S conferring acquired resistance to osimertinib. J Int Med Res. (2020) 48:300060520927918. doi: 10.1177/0300060520927918 32600081 PMC7328482

[7] Tang K Jiang N Kuang Y He Q Li S Luo J . Overcoming T790M mutant small cell lung cancer with the third-generation EGFR-TKI osimertinib. Thorac Cancer. (2019) 10:359–64. doi: 10.1111/1759-7714.12927 PMC6360229 30521113

[8] Oser M Niederst M Sequist L Engelman J . Transformation from non-small-cell lung cancer to small-cell lung cancer: molecular drivers and cells of origin. Lancet Oncol. (2015) 16:e165–72. doi: 10.1016/S1470-2045(14)71180-5 PMC4470698 25846096

[9] Fitzmaurice C . Global, regional, and national cancer incidence, mortality, years of life lost, years lived with disability, and disability-adjusted life-years for 32 cancer groups, 1990 to 2015: A systematic analysis for the global burden of disease study. JAMA Oncol. (2017) 3:524–48. doi: 10.1001/jamaoncol.2016.5688 PMC6103527 27918777

[10] He L Dar A . Targeting drug-resistant mutations in ALK. Nat Cancer. (2022) 3:659–61. doi: 10.1038/s43018-022-00390-1 PMC9919885 35726064

[11] Solomon BJ Bauer TM Ignatius Ou SH Liu G Hayashi H Bearz A . Post hoc analysis of lorlatinib intracranial efficacy and safety in patients with ALK-positive advanced non-small-cell lung cancer from the phase III CROWN study. J Clin Oncol. (2022) 40:3593–602. doi: 10.1200/JCO.21.02278 PMC9622589 35605188

[12] Lingling X Maoxi C Wei Y Jieting Z Yuanyuan Y Ning X . Transformation of NSCLC to SCLC harboring EML4-ALK fusion with V1180L mutation after alectinib resistance and response to lorlatinib: A case report and literature review. Lung Cancer (Amsterdam Netherlands). (2023) 186:107415. doi: 10.1016/j.lungcan.2023.107415 37907052

[13] Ou S Lee T Young L Fernandez-Rocha M Pavlick D Schrock A . Dual occurrence of ALK G1202R solvent front mutation and small cell lung cancer transformation as resistance mechanisms to second generation ALK inhibitors without prior exposure to crizotinib. Pitfall of solely relying on liquid re-biopsy? Lung Cancer (Amsterdam Netherlands). (2017) 106:110–4. doi: 10.1016/j.lungcan.2017.02.005 28285684

[14] Lai L Meng W Wei J Zhang X Tan Z Lu Y . Transformation of NSCLC to SCLC after 1st- and 3rd-generation EGFR-TKI resistance and response to EP regimen and erlotinib: 2 CARE-compliant case reports. Medicine. (2021) 100:e25046. doi: 10.1097/MD.0000000000025046 33725888 PMC7969239

[15] Offin M Chan J Tenet M Rizvi H Shen R Riely G . Concurrent RB1 and TP53 Alterations Define a Subset of EGFR-Mutant Lung Cancers at risk for Histologic Transformation and Inferior Clinical Outcomes. J Thorac Oncol Off Publ Int Assoc Study Lung Cancer. (2019) 14:1784–93. doi: 10.1016/j.jtho.2019.06.002 PMC6764905 31228622

[16] Mc Leer A Foll M Brevet M Antoine M Novello S Mondet J . Detection of acquired TERT amplification in addition to predisposing p53 and Rb pathways alterations in EGFR-mutant lung adenocarcinomas transformed into small-cell lung cancers. Lung Cancer (Amsterdam Netherlands). (2022) 167:98–106. doi: 10.1016/j.lungcan.2022.01.008 35183375

[17] Li J Wei B Feng J Wu X Chang Y Wang Y . Case report: TP53 and RB1 loss may facilitate the transformation from lung adenocarcinoma to small cell lung cancer by expressing neuroendocrine markers. Front Endocrinol. (2022) 13:1006480. doi: 10.3389/fendo.2022.1006480 PMC9792468 36583000

[18] Niederst M Sequist L Poirier J Mermel C Lockerman E Garcia A . RB loss in resistant EGFR mutant lung adenocarcinomas that transform to small-cell lung cancer. Nat Commun. (2015) 6:6377. doi: 10.1038/ncomms7377 25758528 PMC4357281

[19] Gazeu A Aubert M Pissaloux D Lantuejoul S Pérol M Ikhlef N . Small-cell lung cancer transformation as a mechanism of resistance to pralsetinib in RET-rearranged lung adenocarcinoma: A case report. Clin Lung Cancer. (2023) 24:72–5. doi: 10.1016/j.cllc.2022.10.005 36437214

[20] Marcoux N Gettinger S O'Kane G Arbour K Neal J Husain H . EGFR-mutant adenocarcinomas that transform to small-cell lung cancer and other neuroendocrine carcinomas: clinical outcomes. J Clin Oncol Off J Am Soc Clin Oncol. (2019) 37:278–85. doi: 10.1200/JCO.18.01585 PMC7001776 30550363

[21] Yu L Bazhenova L Gold K Tran L Hilburn V Vu P . Clinicopathologic and molecular characteristics EGFR-mutant lung adenocarcinomas that transform to small cell lung cancer after TKI therapy. Trans Lung Cancer Res. (2022) 11(3):452–61. doi: 10.21037/tlcr-21-665 PMC8988081 35399568

[22] Sacks D Baxter B Campbell B Carpenter J Cognard C Dippel D . Multisociety consensus quality improvement revised consensus statement for endovascular therapy of acute ischemic stroke. Int J stroke Off J Int Stroke Soc. (2018) 13:612–32. doi: 10.1177/1747493018778713 29786478

[23] Liang X Lin A Wang Q Zhang J Luo P Cell plasticity in patients with NSCLC: The controversial origins of transformed SCLC. Biomed. pharmacother. = Biomed. pharmacotherapie. (2022) 149:112909. doi: 10.1016/j.biopha.2022.112909 36068773

[24] Liu S Reck M Mansfield A Mok T Scherpereel A Reinmuth N . Updated overall survival and PD-L1 subgroup analysis of patients with extensive-stage small-cell lung cancer treated with atezolizumab, carboplatin, and etoposide (IMpower133). J Clin Oncol Off J Am Soc Clin Oncol. (2021) 39:619–30. doi: 10.1200/JCO.20.01055 PMC8078320 33439693

[25] Goldman J Dvorkin M Chen Y Reinmuth N Hotta K Trukhin D . Durvalumab, with or without tremelimumab, plus platinum-etoposide versus platinum-etoposide alone in first-line treatment of extensive-stage small-cell lung cancer (CASPIAN): updated results from a randomised, controlled, open-label, phase 3 trial. Lancet Oncol. (2021) 22:51–65. doi: 10.1016/S1470-2045(20)30539-8 33285097

[26] Ma B Zhou Y Shang Y Zhang Y Xu B Fu X . Sintilimab maintenance therapy post first-line cytokine-induced killer cells plus chemotherapy for extensive-stage small cell lung cancer. Front Oncol. (2022) 12:852885. doi: 10.3389/fonc.2022.852885 36158690 PMC9507303

[27] Ma S He Z Liu Y Wang L Yang S Wu Y . Sintilimab plus anlotinib as second or further-line therapy for extensive disease small cell lung cancer: a phase 2 investigator-initiated non-randomized controlled trial. EClinicalMedicine. (2024) 70:102543. doi: 10.1016/j.eclinm.2024.102543 38516099 PMC10955204

[28] Yuan G Liu X Zhang X Song W Lu J Ding Z . Remarkable response to PD-1 inhibitor in a patient with extensive-stage small cell lung cancer: a case report and literature review. Front Immunol. (2023) 14:1267606. doi: 10.3389/fimmu.2023.1267606 37781394 PMC10537924

[29] Zhang Y Li X Tang Y Xu Y Guo W Li Y . Rapid increase of serum neuron specific enolase level and tachyphylaxis of EGFR-tyrosine kinase inhibitor indicate small cell lung cancer transformation from EGFR positive lung adenocarcinoma? Lung Cancer (Amsterdam Netherlands). (2013) 81:302–5. doi: 10.1016/j.lungcan.2013.04.005 23683536

