# Supplementary material for: ALK-rearranged and EGFR wild-type lung adenocarcinoma transformed to small cell lung cancer: a case report
Source: Front Oncol. 2024 Apr 23;14:1395654. doi: 10.3389/fonc.2024.1395654 (PMC11078020; doi:10.3389/fonc.2024.1395654)
Supplement: Supplementary file 1 [file Table_1.docx]

**Table 1. Treatment detail of SCLC transformation patient.**

| Sex | Male |
| --- | --- |
| Age | 47 |
| Smoking history | Yes |
| Surgery | Complete resection |
| Adjuvant Chemotherapy (CT) | Yes |
| CT regimen | Pemetrexed and Cisplatin (four cycles) |
| Adjuvant radiotherapy | No |
| Target therapy for advanced stage | Crizotinib, TGRX-326, Loratinib |
| Duration of target therapy | 27 months |
| Treatment options after transformed to SCLC | Etoposide + Carboplatin + Sintilimab (four cycles) |
| Current treatment options | Albumin-bound paclitaxel and anlotinib |
