# Supplementary figures and images for: ALK-rearranged and EGFR wild-type lung adenocarcinoma transformed to small cell lung cancer: a case report
Source: Front Oncol. 2024 Apr 23;14:1395654. doi: 10.3389/fonc.2024.1395654 (PMC11078020; doi:10.3389/fonc.2024.1395654)

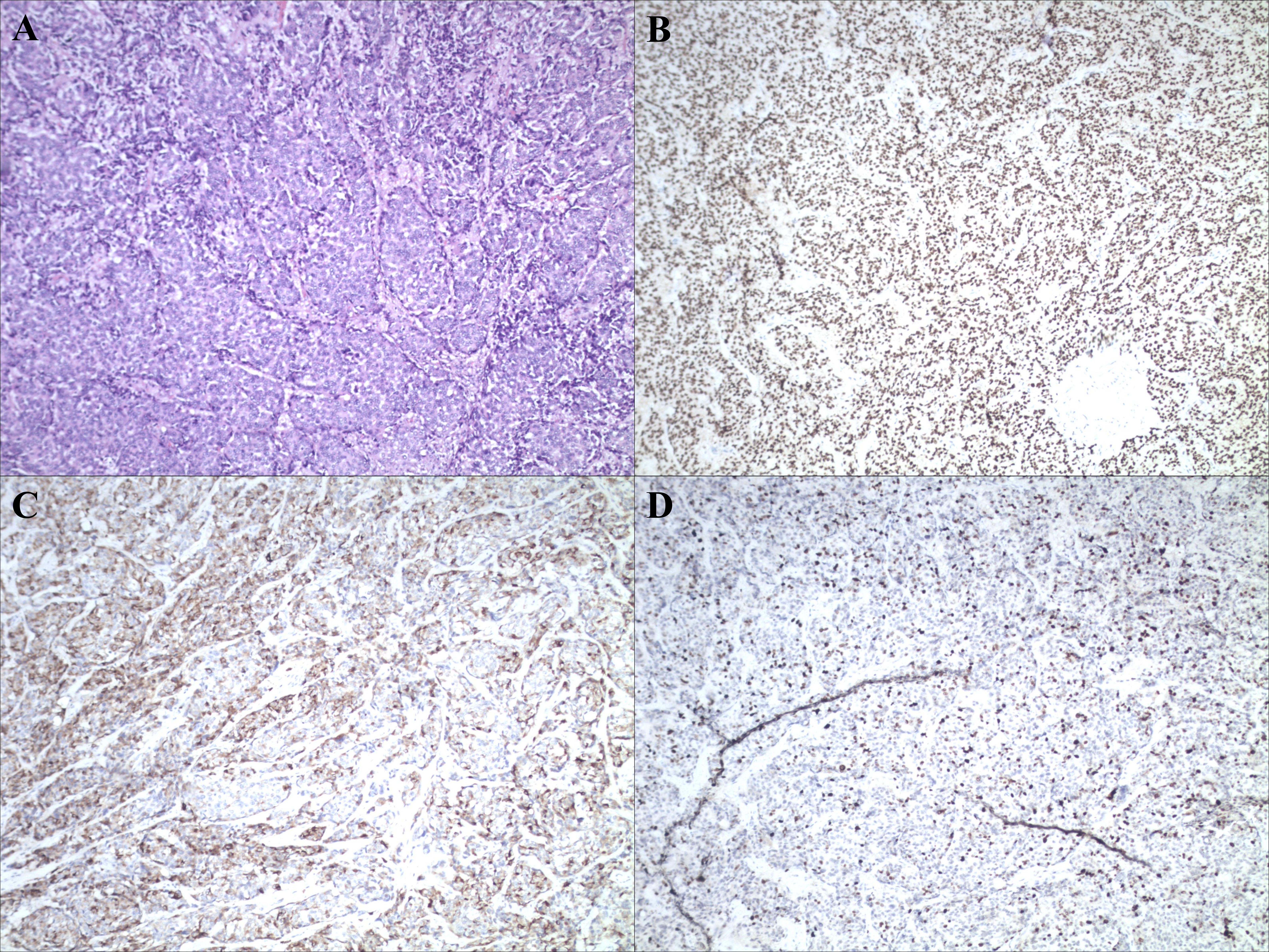

Supplement: Supplementary Figure 1 — H&E staining and IHC staining of surgical resection specimens. (A) H&E staining of surgically excised specimens. (B-D) IHC staining of the surgically resected specimen showed positivity for TTF-1, CK, and Ki67. [file Image_1.jpeg]

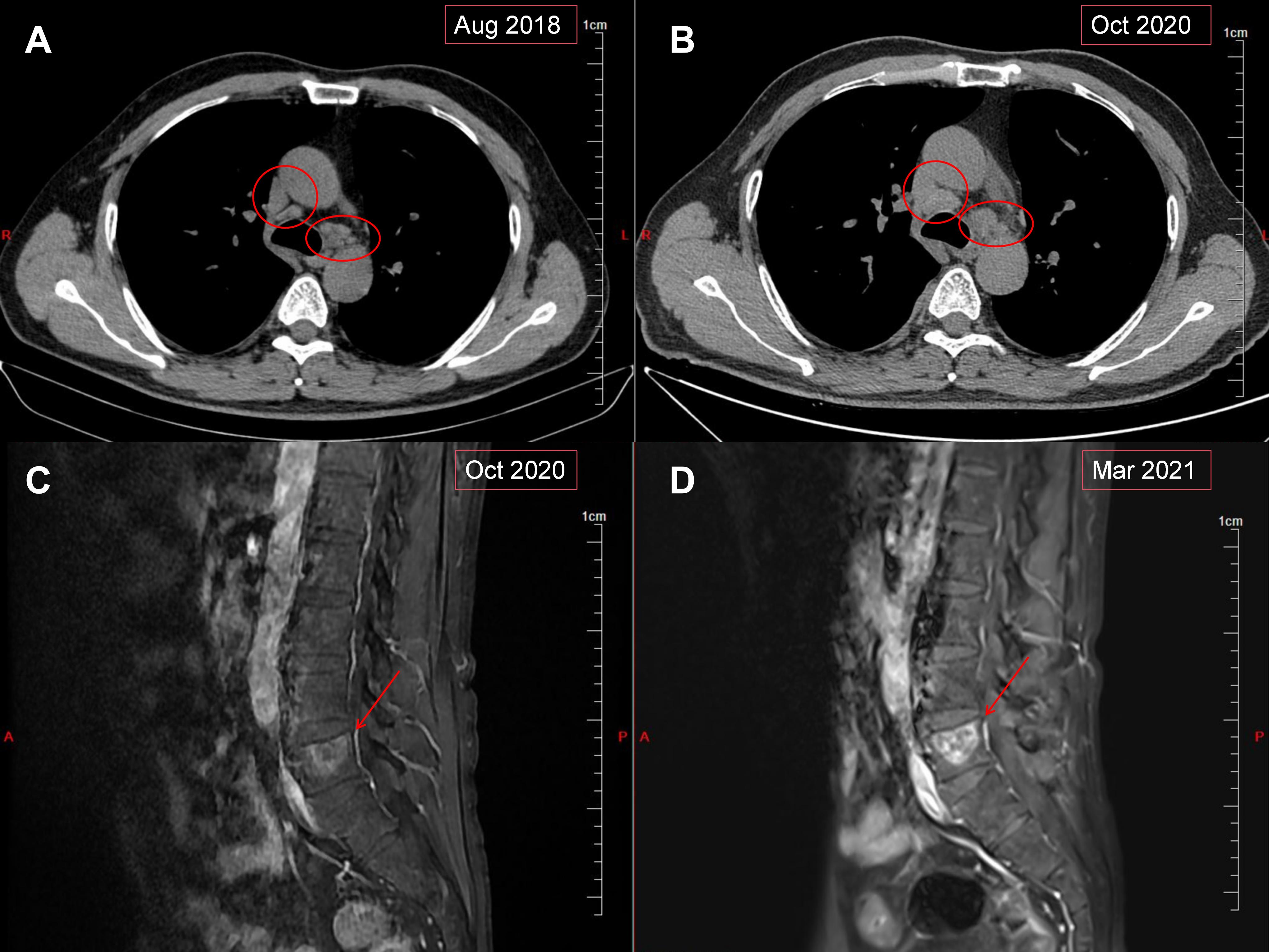

Supplement: Supplementary Figure 2 — CT images of the patient’s chest and MRI images of the abdomen. (A, B) Mediastinal window of chest CT demonstrating the patient’s mediastinal lymph nodes. (C, D) Abdominal MRI images showed tumor invasion of the patient’s lumbar spine at L4. [file Image_2.jpeg]

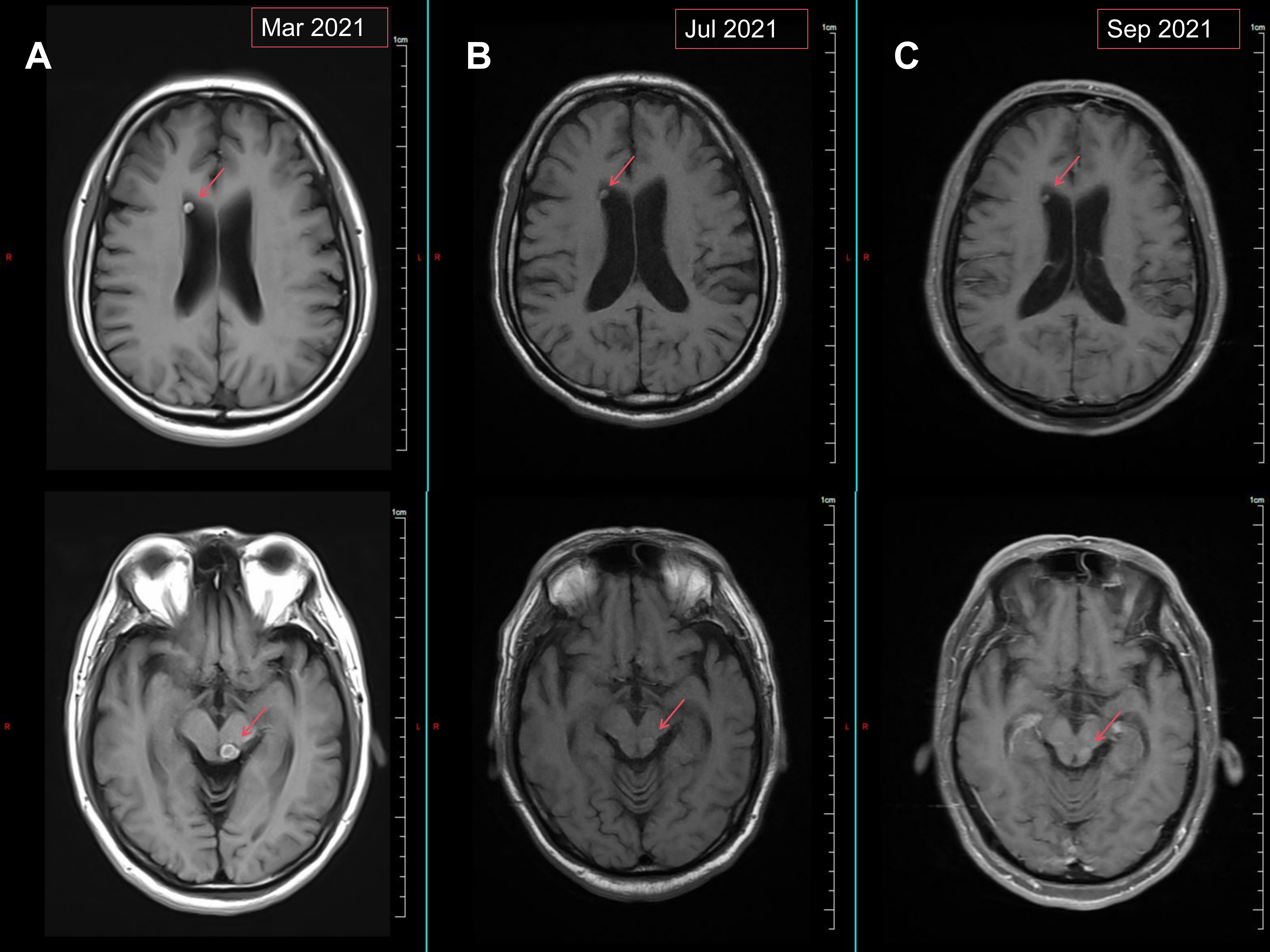

Supplement: Supplementary Figure 3 — MRI image of the patient’s cranium. (A). Cranial MRI images of the patient before whole brain radiotherapy. (B, C) are cranial MRI images of the patient after whole brain radiotherapy. [file Image_3.jpeg]
